# New records of the rare calcareous sponge *Paragrantia waguensis* Hôzawa, 1940

**DOI:** 10.3897/zookeys.546.6122

**Published:** 2015-12-16

**Authors:** Rob W.M. Van Soest, Bert W. Hoeksema, James D. Reimer, Nicole J. De Voogd

**Affiliations:** 1 Naturalis Biodiversity Center, Dept. Marine Zoology, P.O. Box 9617, 2300 RA Leiden, The Netherlands; 2 University of the Ryukyus, Dept. Biology, Chemistry & Marine Sciences, 1 Senbaru, Nishihara, Okinawa, Japan 903-0213

**Keywords:** Porifera, Calcarea, Leucosolenida, Grantiidae, Japan, apopylar spicules

## Abstract

*Paragrantia waguensis* Hôzawa is reported from coastal reefs of the island of Okinawa. This rare species was previously known only from Central Japan, Mie Prefecture. It has peculiar apopylar tetractine spicules, so far unique among Calcarea. We present in situ images of the species and a full description including SEM images of skeletal structure and spicule complement. The status of *Paragrantia* as a separate genus of the family Grantiidae distinct from *Grantia* Fleming is confirmed on the basis of a morphological and molecular comparison with the European type species of *Grantia*, *Grantia compressa* (Fabricius).

## Introduction

We report here the recent collection (2006, 2014) of a rare and curious calcareous sponge species from Okinawa, *Paragrantia waguensis* Hôzawa (1940), at considerable distance (1500 km) from its type locality in eastern mid-Japan (Mie Prefecture). The species was first collected in Okinawa in 2006 and mentioned briefly in a paper reporting its chemistry (Tianero et al. 2009). Subsequently (2014), one of us (BWH) collected the same species slightly to the north of the 2006 locality.

*Paragrantia* Hôzawa (1940) was erected as a new genus in the family Grantiidae Dendy (1893) (Porifera, Calcarea, Calcaronea, Leucosolenida) on the basis of a curious structure in the exhalant openings of its type species, *Paragrantia waguensis*. The genus and its type species were not seriously evaluated since they were described in 1940: the genus was assigned to the synonymy of the genus *Scypha* Gray (1821) in Burton’s literature review of the Calcarea (1963: 448) and the type species was considered a junior synonym of the boreal widespread species *Scypha compressa* (Fabricius, 1780) (= *Grantia compressa*), meriting only the comment that its general appearance resembled that of the well-known *Grantia compressa*. Burton (*l.c.*) simply summarized Hôzawa’s description as a ‘named form’ under the synonyms of *Scypha compressa*, in the process omitting to provide information on its diactines (see below). Burton’s (1963) ‘decisions’ about the classification of the Calcarea were severely criticized at the time, and his synonymy assignments were generally considered unacceptable.

The major revision of the Calcarea presented in the framework of the Systema Porifera project (Borojevic et al. 2002a, b, c) ignored the genus entirely, as was the case in the published preview of the subclass Calcaronea of Borojevic et al. (2000). Only in the ‘Annotated List of unrecognizable sponge taxa and unavailable names’ added as an appendix to the Systema Porifera (Hooper and Van Soest 2002: 1701–1706) the name *Paragrantia* is mentioned as a ‘possible synonym of *Grantia*’. The World Porifera Database (Van Soest et al. 2015) assigned the species on that basis to the ‘accepted’ combination *Grantia waguensis*.

Despite Hôzawa’s excellent description and generally good illustrations of *Paragrantia waguensis*, we feel induced by its subsequent lack of proper evaluation, to once again draw attention to it. Here, we fully describe our specimens, providing in situ photos, compare it with Hôzawa’s description (unfortunately the type material itself, kept in the Tôhoku University Museum (TUMC), was not available to us), and pose the question of the validity of the genus *Paragrantia*. For that purpose, we compared our specimens with a representative sample of the type species of the genus *Grantia*, *Grantia compressa* (Fabricius, 1780) and performed a molecular sequence analysis of the two species and some of its assumed relatives.

## Methods

Specimens were collected using SCUBA by J. Tanaka (University of the Ryukyus) in 2006, and by BWH, as a guest of JDR, in 2014, subsequently identified by NJDV and RWMVS, and registered in the RMNH collection. For our comparison with *Grantia compressa*, we used a specimen from Roscoff, W coast of France, incorporated in the ZMA collection, now housed in Naturalis Biodiversity Center.

Specimens were sectioned by hand: cross sections perpendicular to the surface and tangential sections of the outer surface (cortical region), the inner surface (atrial region), and the oscular fringe. The sections were air dried, mounted on stubs, and sputter coated for examination under SEM. Spicules were dissociated using household bleach, washed five times in distilled water, and subsequently plated on glass slides for light-microscopic measurements and on SEM stubs for examination and micrographing under SEM.

Measurements of the spicules are given as smallest-*average*-largest of 25 spicules of each distinct type.

To verify the conclusions from the morphological comparison of *Paragrantia waguensis* with *Grantia compressa*, a 28S rDNA sequence (430 bp) of *Paragrantia waguenis* (sample RMNH Por. 9317) was provided by the Naturalis Barcode Laboratory. DNA was extracted using the NucleoMag 96 Tissue kit by Macherey-Nagel on a Thermo Scientific Kingfisher Flex magnetic bead extraction robot with a final elution volume of 150 µl. The forward and reverse of the C2-D2 region of the nuclear ribosomal 28S was amplified (Forward primer 5’GAAAAGAACTTTGRARAGAGAGT 3’ and Reverse primer 5’TCCGTGTTTCAAGACGGG 3’). Template was diluted ten times before amplification and added with 18.8 µl of ultrapure MQ water, 2.5 µl PCR buffer, 0.5 µl dNTP (containing 2.5 mM) and 0.25 µl Taq (5 units per µl) to a total reaction volume of 25 µl. PCR cycling consisted of an initial denaturation step at 94 °C for 3 mins, followed by 40 cycles each consisting of 15 secs at 95 °C, 30 secs at 50 °C, 40 secs at 72 °C, and a final extension of 5 mins at 72 °C. Bidirectional sequencing was performed at BaseClear (http://www.baseclear.com/). Sequences were edited manually with Sequencher 4.10.1 (Gene Codes Corporation).

With the obtained sequence we performed a BLAST analysis provided by the NCBI website (http://blast.ncbi.nlm.nih.gov/Blast.cgi), and downloaded a representative set of partial 28S sequences of calcaronean species showing up in the BLAST result. The set of sequences included a sequence of *Grantia compressa* provided by Manuel et al. (2004), 14 other available Leucosolenida-sequences submitted by various research groups belonging to Grantiidae, Sycettidae, Jenkinidae, Amphoriscidae and Lelapiidae. We added as outgroup sequence the calcinean *Pericharax heteroraphis* (recently revised and renamed as *Pericharax orientalis* Van Soest & De Voogd, 2015). The combined dataset of 17 sequences (see Table 1) was then aligned using ClustalW, trimmed to approximately equal numbers of basepairs (407 bp), and subsequently analyzed phylogenetically, using the MEGA package vs 06.6 for Mac (http://www.megasoftware.net/megamac.php). For the phylogeny reconstruction we chose the Maximum Likelihood statistical method with a Bootstrap method set at 100 replicates. As Substitution Model we chose the Tamura-Nei model and – based on model testing algorithm in MEGA – we used GTR+G as Evolutionary Model. Further parameters were used in their default settings.

**Table 1.** Leucosolenida species of which partial 28S sequences were downloaded from the NCBI website (http://www.ncbi.nlm.nih.gov/) and were used for an evaluation of the phylogenetic relationships of *Paragrantia waguensis* and *Grantia compressa*. From left to right columns list genus, species and family names, accession numbers of the sequences, and literature sources. The results of the phylogenetic analysis are represented in Fig. 8.

Genus	Species	Family	Accession number	Source
*Paragrantia*	*waguensis*	Grantiidae	KT277668.1	present study
*Grantia*	*compressa*	Grantiidae	AY563538.1	Manuel et al. 2004
*Ute*	*ampullacea*	Grantiidae	JQ272226.1	Voigt et al. 2012
*Leucandra*	*nicolae*	Grantiidae	JQ272268.1	Voigt et al. 2012
*Leucandra*	*aspera*	Grantiidae	AY563535.1	Manuel et al. 2004
*Leucandra*	sp.	Grantiidae	JQ272265.1	Voigt et al. 2012
*Aphroceras*	sp.	Grantiidae	AM181001.1	Dohrmann et al. 2006
*Teichonopsis*	*cylindrica*	Grantiidae	JQ272264.1	Voigt et al. 2012
*Synute*	*pulchella*	Grantiidae	JQ272274.1	Voigt et al. 2012
*Sycon*	*capricorn*	Sycettidae	AM181000.1	Dohrmann et al. 2006
Scypha (=Sycon)	*raphanus*	Sycettidae	AY563537.1	Manuel et al. 2004
*Anamixilla*	*torresi*	Jenkinidae	AY563536.1	Manuel et al. 2004
*Leucascandra*	*caveolata*	Jenkinidae	JQ272259.1	Voigt et al. 2012
*Paraleucilla*	*magna*	Amphoriscidae	JQ272267.1	Voigt et al. 2012
*Paraleucilla*	sp.	Amphoriscidae	AY563540.1	Manuel et al. 2004
*Grantiopsis*	*heroni*	Lelapiidae	AY563539.1	Manuel et al. 2004

The systematic classification generally follows the Systema Porifera (Hooper and Van Soest 2002), chapter on Leucosolenida (Borojevic et al. 2002b).

## Results

### Phylum Porifera Class Calcarea Subclass Calcaronea Order Leucosolenida Family Grantiidae Genus *Paragrantia*

#### Paragrantia waguensis

Taxon classificationAnimaliaLeucosolenidaGrantiidae

Hôzawa, 1940

**Figure 1.**
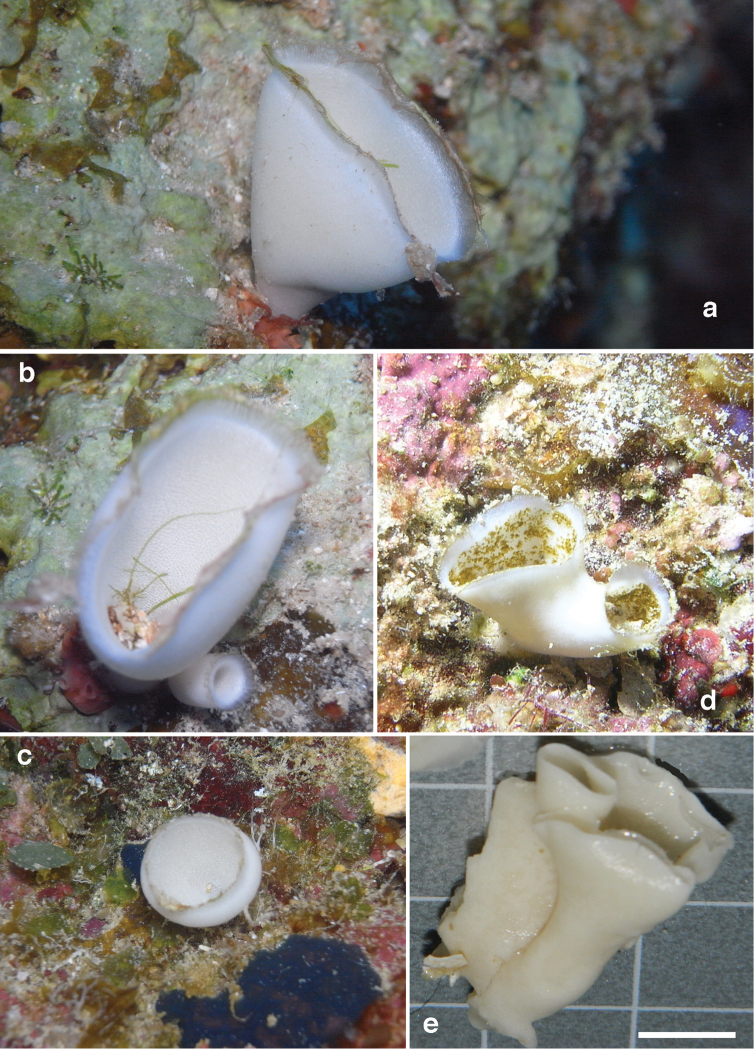
Habitus of *Paragrantia waguensis*. **a** *in situ* photo of oval cup-shaped individual at Manza, Okinawa (photo B.W. Hoeksema of RMNH Por. 9317) **b** ditto and small tubular rounded bud (photo B.W. Hoeksema) **c** ditto of small rounded cup-shaped individual (B.W. Hoeksema) **d** budded individual showing mottled algal growth, from Onna village, Okinawa (photo J. Tanaka) **e** preserved specimens from Manza, RMNH Por. 3901) (scale bar = 1 cm).

**Figure 2.**
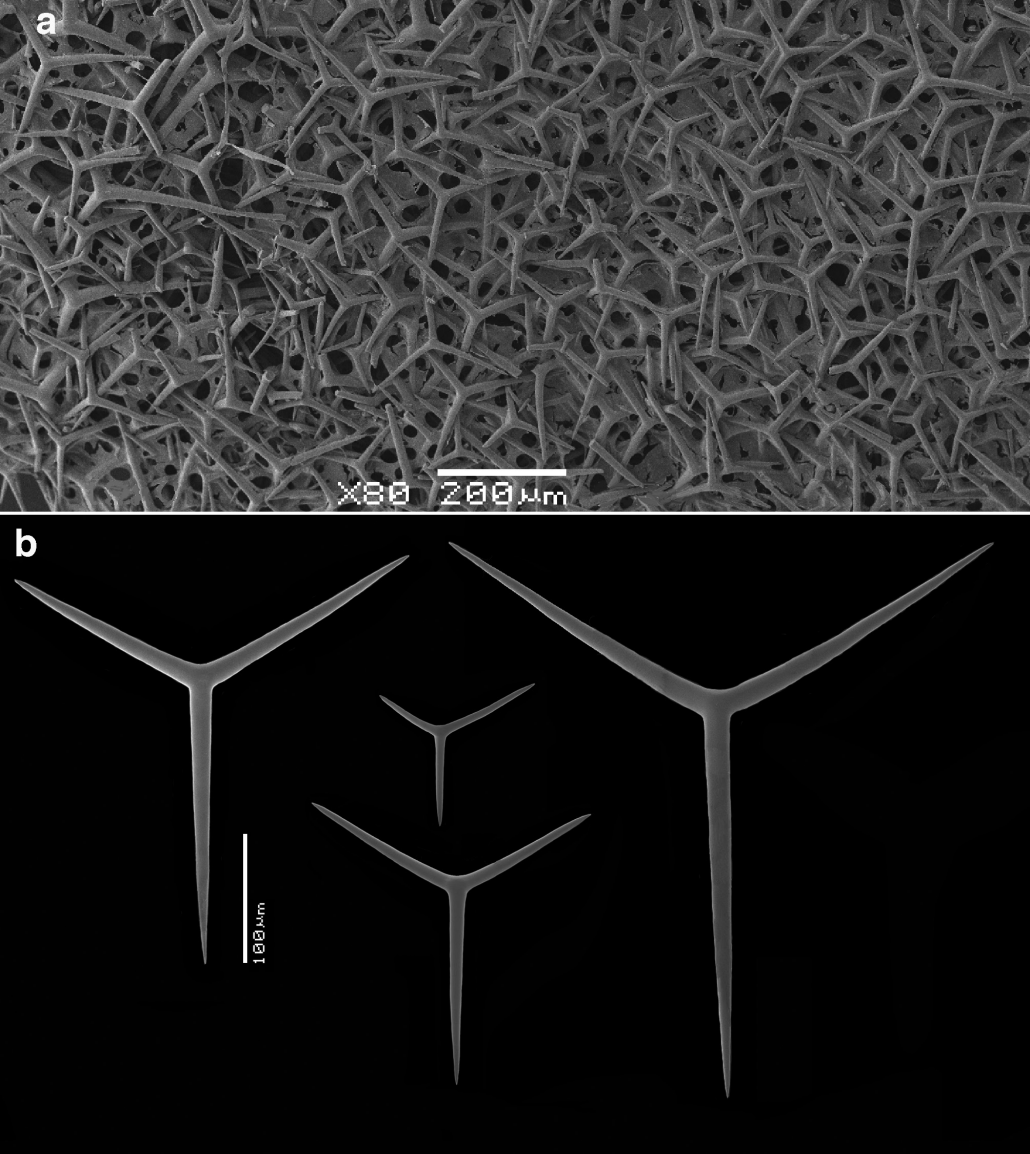
SEM images of cortical region of *Paragrantia waguensis* and its cortical spicules. **a** overview of cortical surface showing dense layer of cortical triactines **b** various sizes and shapes of cortical triactines.

**Figure 3.**
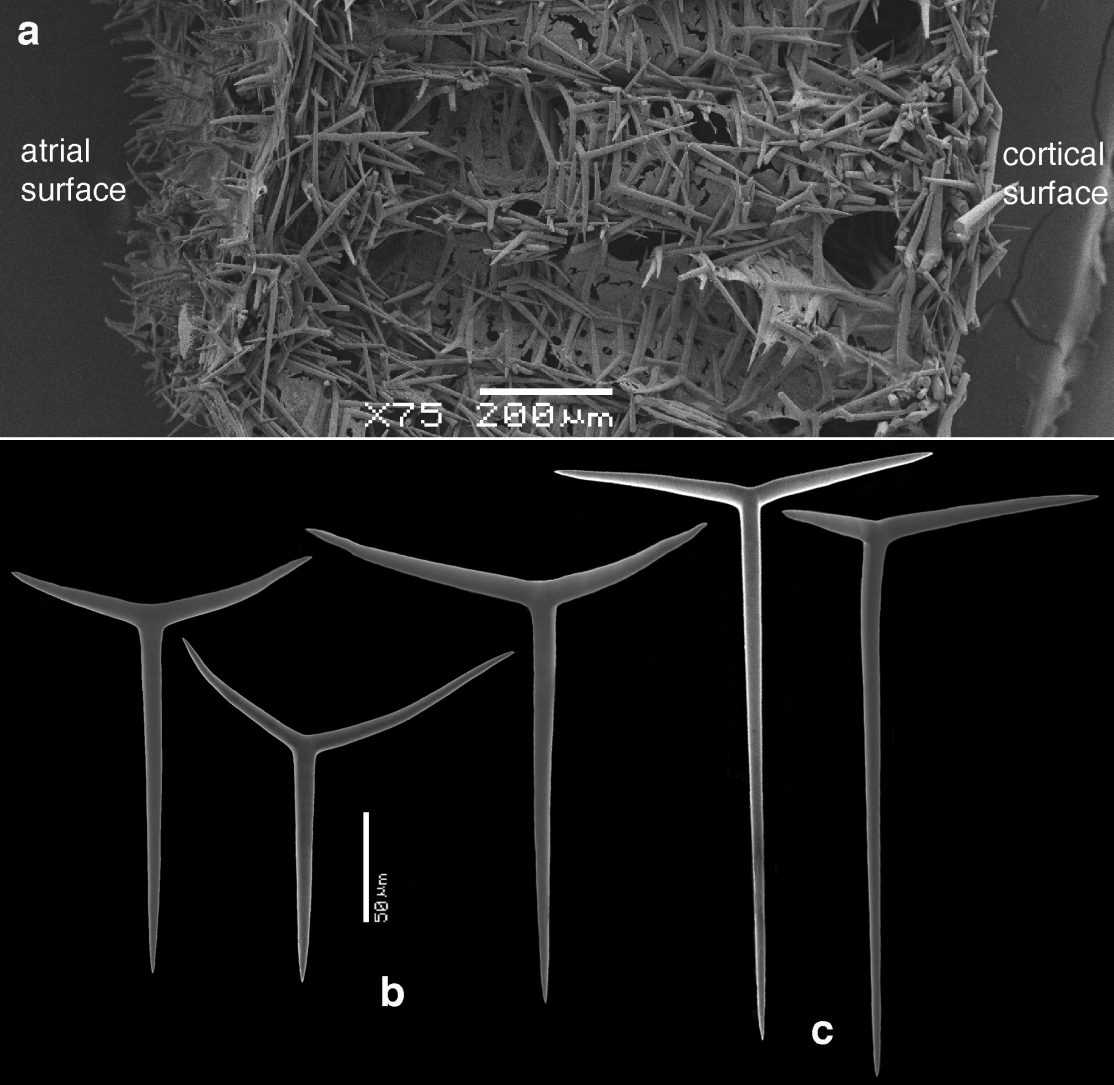
SEM images of cross section of *Paragrantia waguensis* and its choanosomal spicules. **a** overview of cross section showing skeleton of tubar chambers and subatrial skeleton **b** various sizes and shapes of tubar triactines **c** subatrial triactines.

**Figure 4.**
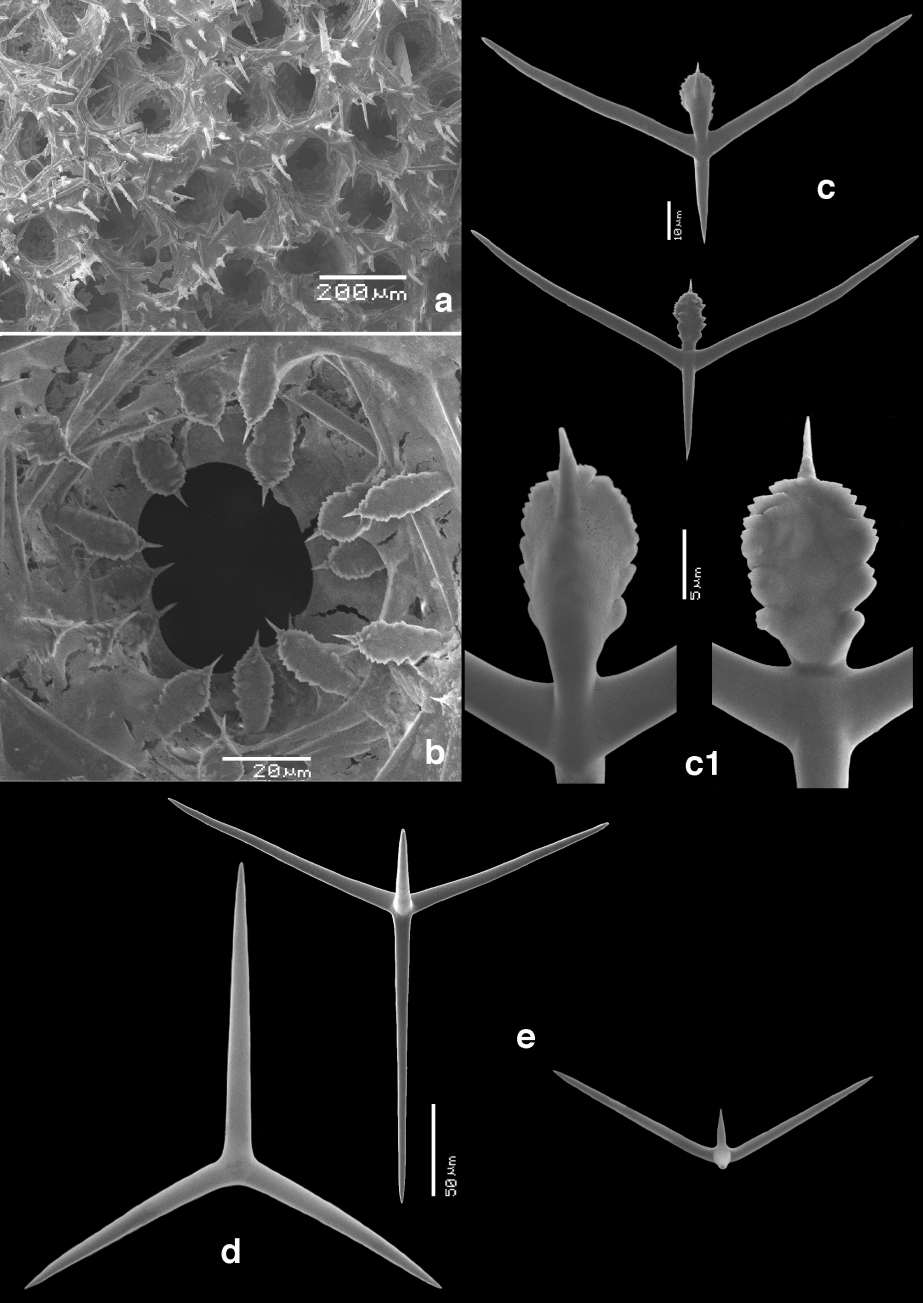
SEM images of atrial region of *Paragrantia waguensis* and its atrial spicules. **a** overview of atrial surface with atrial chambers **b** detail of atrial apopylar chambers showing the position of the apopylar spicules **c** apopylar spicules **c1** details of apical actines of **c d** atrial triactine **e** atrial tetractines.

**Figure 5.**
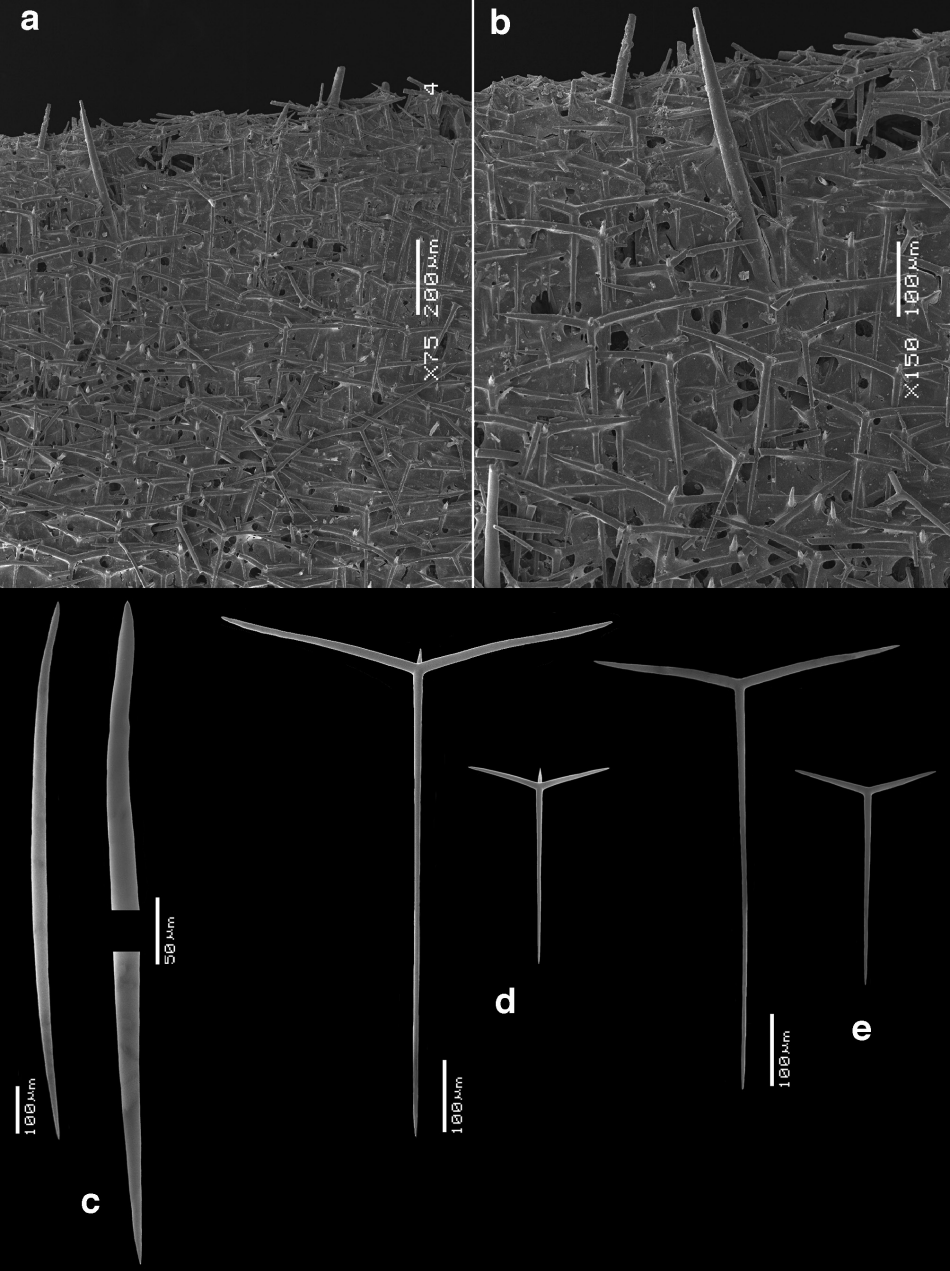
SEM images of the fringe of *Paragrantia waguensis* and its fringe spicules. **a** overview of fringe inner surface **b** detail of the same showing presence and position of the spicules **c** diactines **d** tetractines **e** triactines.

Figs 1 , 2 , 3 , 4 , 5

Paragrantia waguensis Hôzawa, 1940: 40, pl. V figs 8–11, text-fig. 4; Burton 1963: 448, text-fig. 274 as named form of *Scypha compressa* (not: *Spongia compressa* Fabricius, 1780).
Grantia waguensis ; Van Soest et al. 2015 on-line.

##### Material.

Naturalis Biodiversity Center, reg. nr. RMNH Por. 9317 (five individuals), Japan, South Kuroshio ecoregion, Okinawa, Manza, approximately 26.5°N, 127.8°E, vertical rocky wall, 25–30 m, coll. B.W. Hoeksema, 10 August 2014; Naturalis Biodiversity Center, reg.nr. RMNH Por. 3901 (three individuals), Japan, South Kuroshio ecoregion, Okinawa, Onna village, approximately 26.5°N, 127.8°E, coral reef slope, 20–55 m, coll. J. Tanaka, 6 May 2006.

Syntype, 8 specimens (not seen) Tôhoku University Museum, reg.nr. TUMC 110908, Japan, Central Kuroshio ecoregion, Mie Prefecture, Wagu, approximately 34.25°N, 136.8°E, coll. S. Hôzawa, July 1933.

##### Description.

Cup-shaped or tubular specimens (Fig. 1a–e), usually being a single rounded ‘person’ in life, but larger individuals may be somewhat elliptical, and occasionally consisting of two or three budded individuals. The cups or tubes have a narrow attachment to the substratum but there is no clear stalk. Outer surface pearly white and smooth, without any visible inhalant structures. The rim is pale purple in color and distinctly fringed. Algae or detritus may stick to the rim. Inside the cup, most specimens are likewise smooth and white, but one of the specimens is mottled greenish due to encrusting algae growing on its inner surface (Fig. 1d). A faint punctate inner surface pattern may be discernible in some individuals, representing the peculiar exhalant chambers characteristic for this species. Size of individuals may vary from 1 to 4 cm in height, 0.5–4 cm in diameter, thickness of the walls up to 1.5 mm. Consistency firm, somewhat flexible, but breakable under pressure. In preserved condition, the shape of the individuals alters notably: the specimens collapse and may become folded and compressed.

**Aquiferous system.** No histological slides were made, but the structure of the skeleton suggests it is syconoid (as was also the case in Hôzawa’s material). There is no evidence of branching choanocyte chambers. Subcortical lacunae are present, regularly distributed and apparently serving as inhalant reservoirs.

**Skeleton of the walls.** (Figs 2–4) In cross section from external side to inside: a fairly thick cortical skeleton of relatively large triactines (Fig. 2a), an articulate tubar skeleton (Fig. 3a) consisting of three or more rows of sagittal triactines with centrifugally directed unpaired actines, subsequently a layer of subatrial strongly sagittal triactines, and an atrial skeleton (Fig. 4a–b) of tetractines and triactines, containing the three-dimensionally rounded atrial exhalant chambers (Fig. 4b) supported and covered by inwardly directed small butterfly-shaped tetractines (Figs 4c–c1) with peculiarly swollen and ornamented apical actines (see below). These small apopylar tetractines in addition to the normal atrial triactines (Fig. 4d) and tetractines (Fig. 4e) are so far unique among the Calcarea.

**Skeleton of the fringe.** (Fig. 5a–b) The main support of the fringe consists of a palisade of long thick diactines tangentially covered outside and inside by sagittal triactines and tetractines differing morphologically from those of the walls.

**Spicules.** Cortical triactines, tubar triactines, subatrial triactines, atrial triactines, atrial tetractines, apopylar tetractines, diactines from the fringe, triactines from the fringe, tetractines from the fringe.

Cortical triactines (Fig. 2b), mostly slightly sagittal, with unpaired actines generally longer than paired actines, but not infrequently all actines are about the same length with the sagittal morphology only expressed by a wider angle between the paired actines; unpaired actines 48–*196.6*–315 × 6–*15.3*–24 µm, paired actines 40–*162.4*–242 × 9–*14.7*–23 µm.

Tubar triactines (Fig. 3b), sagittal, unpaired actines usually longer than paired actines, with paired actines often having a wide angle with the unpaired actines, frequently rather ‘oxhorn shaped’, the ends curved slightly inwards; unpaired actines 93–*195.2*–270 × 8–*13.6*–21 µm, paired actines 92–*148.2*–241 × 9–*13.4*–21 µm.

Subatrial triactines (Fig. 3c), strongly sagittal, with paired actines wide angled, occasionally at right angles with the unpaired actines, but often not in the same plane, unpaired actines much longer than paired actines; unpaired actines 207–*242.3*–291 × 6–*8.3*–10 µm, paired actines 61–*85.0*–111 × 6–*8.4*–10 µm.

Atrial triactines (Fig. 4d), strongly sagittal, with unpaired actines longer than the paired actines, but differentiated from the subatrial triactines by longer paired actines; unpaired actines 150–*247.5*–354 × 8–*9.6*–11 µm, paired actines 114–*171.1*–261 × 7–*10.3*–12 µm.

Atrial tetractines (Fig. 4e), sagittal, with unpaired and paired actines not strongly different in length, but occasionally the paired actines are slightly longer, and apical actines conical; unpaired actines 42–*118.7*–226 × 7–*9.7*–10 µm, paired actines 105–*132.7*–171 × 7–9.0–10 µm, apical actines 30–*37.3*–63 × 6–*8.6*–12 µm.

Atrial exhalant chamber tetractines (apopylar tetractines) (Figs 4c, 4c1), butterfly-shaped, sagittal, with short conical unpaired actines, distinctly longer paired actines, and with the apical actines swollen and ornamented with irregular spines, curved slightly inwards, likened to ‘torches’ by Hôzawa (1940: 42); unpaired actines 14–*23.6*–39 × 3–*3.8*–5 µm, paired actines 27–*64.1*–78 × 3–*4.1*–5 µm; apical actines 13–*19.4*–24 × 5–*8.2*–10 µm.

Diactines (Fig. 5c) from the fringe, fusiform, sharply pointed, 360–*703.2*–990 × 12–*21.6*–29 µm.

Tetractines (Fig. 5d) from the fringe, strongly sagittal, unpaired actines longer than the paired actines, which are widely flaring, and apical actines conical, sharply pointed; unpaired actines 165–*284.8*–528 × 9–*10.6*–15 µm, paired actines 81–*167.6*–273 × 8–*10.3*–12 µm, apical actines 16–*39.3*–76 × 5–*7.2*–10 µm.

Triactines (Fig. 5e) from the fringe, strongly sagittal, with much longer unpaired actines than paired actines (similar to subatrial triactines), 156–*223.5*–279 × 6–*8.8*–10 µm, paired actines 63–*84.8*–105 × 7–*9.2*–10 µm.

##### Ecology.

No data were provided by Hôzawa, but the Okinawa specimens were from a steep reef slope at 20–55 m depth, growing among coralline and turf algae, and encrusting sponges.

##### Distribution.

Warm-temperate (Mie Prefecture) and subtropical (Okinawa) regions of Japan.

## Discussion

### Comparison with Hôzawa’s specimens

The habit of Hozawa’s specimens is described from eight preserved individuals. A photo is given of two specimens (Hôzawa 1940: pl. V fig. 8) having a fusiform shape ending in a terminal oscule. This is unlike our own cup-shaped individuals. However, we assume that like in our own specimens the habit was changed rather dramatically into laterally compressed, partially ‘branched’ individuals after preservation. We here assume that the live habit was likely more cup-shaped/tubular. The Tôhoku University Museum was unable to grant our request for a loan of the type material (e-mail of Mr. Jun Nemoto, technical staff of the Tôhoku University Museum), but we are confident from Hôzawa’s description and illustrations, and the changes we observed in our own material between live and preserved specimens (cf. Fig. 1a–d and Fig. 1e), that the features are sufficiently similar to consider both groups of specimens as belonging to the same species.

The general structure of the skeleton and the overall diversity and sizes of the spicules likewise match closely (as can be observed from Table 2), so microscopical features between the two sets of specimens also confirm that they belong to the same species.

**Table 2.** *Paragrantia waguensis*, spicule size data (micrometers). The data of the type specimens from Wagu (taken from Hôzawa 1941) are compared to those of specimens from Okinawa (our own measurements of RMNH Por. 9317 and 3901 combined), divided into three sets, a. spicules of the cortex and the chamber layer, b. spicules of the atrial region, and c. spicules of the fringe.

a. Spicules of the cortex and the chamber layer								
	cortical triactines		tubar triactines		subatrial triactines			
	unpaired	paired	unpaired	paired	unpaired	paired		
Type specimens: Wagu	80-420 x 8-20	50-220×4-16	140-225×10-16	90-160×10-16	180-350×8-10	65-110×8-10		
Okinawa	48-315×6-24	40-242×9-23	93-270×8-21	92-241×9-21	207-291×6-10	61-111×6-10		
b. Spicules of the atrial region								
	atrial triactines		atrial tetractines			apopylar triactines		
	unpaired	paired	unpaired	paired	apical	unpaired	paired	apical
Type specimens: Wagu	100-390×8-10	70-200×8-10	100-390×10	115-170×10	40-90×12	20-25×4	56-70×4	20×12-18
Okinawa	150-354×8-11	114-261×7-12	42-226×7-10	105-171×7-10	30-63×6-12	14-39×3-5	27-78×3-5	13-24×5-10
c. Spicules of the fringe								
	Fringe diactines	Fringe triactines		Fringe tetractines				
		unpaired	paired	unpaired	paired	apical		
Type specimens: Wagu	300-880×14-28	340×10	150×12	150-420×8	150-200×10	20-40×10		
Okinawa	360-990×12-29	156-279×6-10	63-105×7-10	165-528×9-15	81-273×8-12	16-76×5-10		

Nevertheless, there are a few clear differences:

- The unpaired actines of the atrial tetractines in Hôzawa’s specimens were given as large as up to 390 µm, whereas in our specimens they were only up to 226 µm.
- The apical actines of the apopylar tetractines in Hôzawa’s specimens were given as having a width of 12–18 µm, whereas ours were only 5–10 µm.
- The tetractines of the fringe in Hôzawa’s specimens were smaller and thinner: e.g. unpaired actines were 150–420 × 8 µm, whereas in our specimens these measured 165–528 × 9–15 µm.

We believe that these differences are too small to consider them as evidence for specific distinction.

### Comparison with *Grantia compressa*

By its possession of a cortical skeleton, an articulated choanosomal skeleton, and lack of pseudosagittal spicules, the present species fits the family Grantiidae. In order to decide whether Hôzawa was right in establishing a new genus for his species *waguensis*, it is necessary to know the properties of the other genera of the family. The syconoid aquiferous system and the absence of long longitudinally arranged diactines (except in the fringe) limits the generic relationships of *waguensis* to *Grantia* Fleming (1828), *Sycandra* Haeckel (1872) and *Teichonopsis* Dendy & Row (1913). All three share the general structure of the skeleton with the present species. The latter two genera are monotypic and are distinguished on unique features, the presence of a special atrial network of tissue strands supported by small diactines (*Sycandra*), or an elaborate shape (*Teichonopsis*). *Sycandra utriculus* (Schmidt, 1869) has a similar structure and spiculation as our specimens (and many *Grantia* species), but the peculiar atrial network forms a unique distinction. *Teichonopsis labyrinthica* (Carter, 1878), vaguely resembles preserved specimens of the present species, but the spiculation differs clearly by the lack of any tetractines and the presence of brushes of small oxeas on the cortical and atrial surfaces. *Grantia* itself, in contrast, has approximately 40 accepted species (Van Soest et al. 2015), with considerable variability of habit and skeletal characters. In order to be able to judge whether the unique features of *waguensis* merit a separate genus status like *Sycandra utriculus* and *Teichonopsis labyrinthica*, or whether it can be assigned to *Grantia* s.l., we here compare our observations on *waguensis* with those of the type species of *Grantia* (and indeed the type of the family Grantiidae), *Grantia compressa*, the well known Purse Sponge of intertidal rocky coasts of Northern Europe. Remarkably, neither the Systema Porifera, chapter on Leucosolenida (Borojevic et al. 2002b), nor its preview publication (Borojevic et al. 2000), presented a proper description and illustration of this important species. We chose a specimen from Roscoff, W coast of France (intertidal, coll. D.A.G. Buizer, February 1977), in the collections of Naturalis Biodiversity Center, reg. nr. ZMA Por. 04159, as our object for comparison.

**Figure 6.**
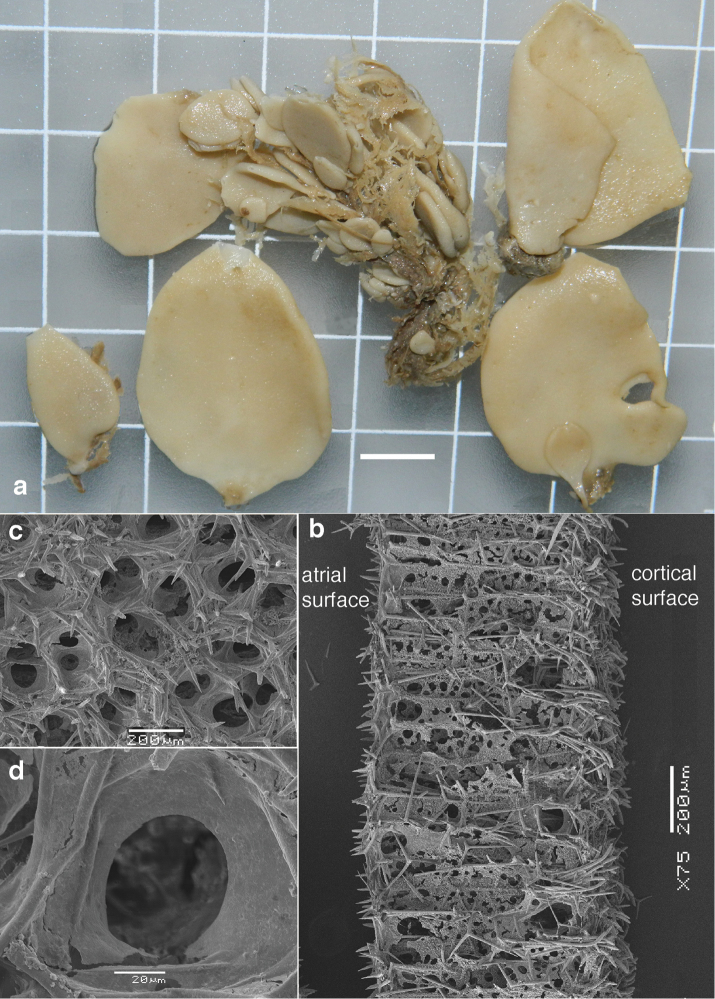
Habitus and skeleton of *Grantia compressa* from Roscoff, W coast of France (ZMA Por. 04159). **a** preserved specimens showing leaf-like habitus (scale bar = 1 cm) **b** SEM image of cross section of the leaf-like wall, showing the cortical brushes of club-shaped diactines and cortical triactines, rows of tubar triactines ending near the atrial region with subatrial triactines, closed off by mixed layer of atrial triactines and tetractines with the apical actines of the tetractines protruding beyond the atrial surface **c** overview of atrial surface and atrial chambers with protruding apical actines of the atrial tetractines **d** detail of atrial chamber lacking differentiated spicules.

**Figure 7.**
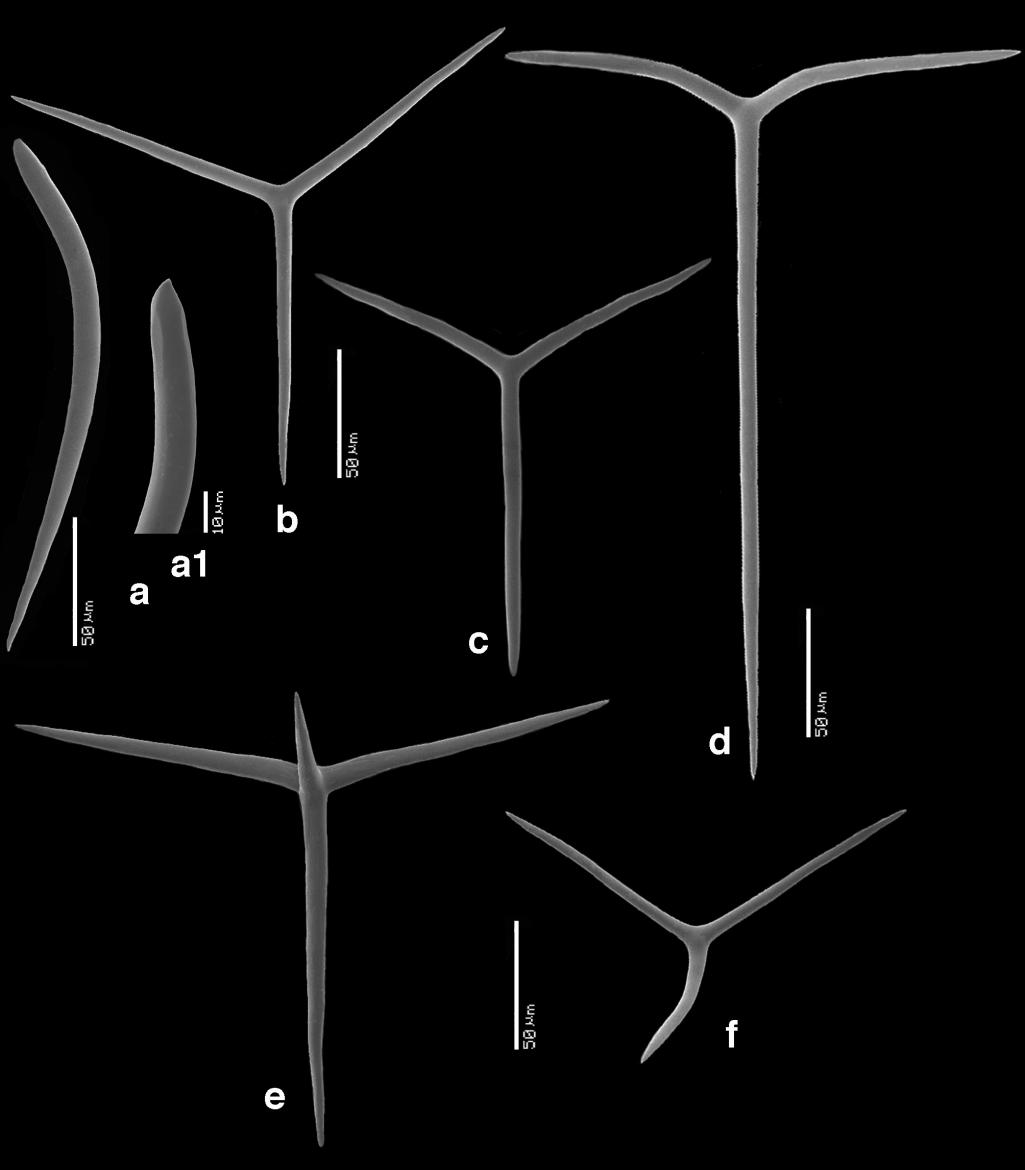
SEM images of the spicules of *Grantia compressa* (ZMA Por. 4159, from Roscoff, W coast of France). **a** club-shaped cortical diactine **a1** detail of head **b** cortical triactine **c** tubar triactine **d** subatrial triactine, e. atrial tetractines, f. atrial triactine.

The sample we studied consisted of a cluster (Fig. 6a) of smaller and larger oval, laterally flattened, individuals, 0.5–4 cm in largest dimension, less than 0.5 cm thick, with terminal small oscules without visible rim. Color off-white, both in situ and in preserved condition. No change in shape when preserved. Skeleton (Fig. 6b) as usual for the family consisting of a cortex, tubar skeleton, and atrial skeleton. The cortical skeleton contains clusters or bouquets of club-shaped diactines overlying a thin (?single) layer of triactines. The tubar skeleton is very regular, made up of a row of sagittal triactines arranged with the unpaired angle pointing outwards. Subatrial triactines are overlying the atrial skeleton. There are rounded lacunae both subdermally and subatrially. The atrial skeleton is made up of atrial tetractines and triactines in variable proportions, the apical actines of the tetractines protrude into the atrial lumen. Apopyles do not have special spicules or skeletal specialization (Figs 6c–d). Spicules (Fig. 7) include cortical diactines (Figs 7a, a1) of 150–350 × 11–15 µm, cortical triactines (Fig. 7b) 88–140 × 7–10 µm, tubar triactines (Fig. 7c) 165–200 × 7–9 µm (unpaired) and 88–102 × 7.5–9 µm (paired), subatrial triactines (Fig. 7d) 255–325 × 9–10 µm (unpaired) and 115–168 × 9–10 µm (paired), atrial tetractines (Fig. 7e) 110–128 × 7.5–10 µm (unpaired), 76–118 × 7–9 µm (paired) and 25–105 × 7–11 µm (apical), and atrial triactines (Fig. 7f) 48–108 × 7.5–10 µm (unpaired) and 102–126 × 8–9 µm (paired).

*Grantia compressa* and *Paragrantia waguensis* differ in the following features:

- In life, *Grantiacompressa* is a flattened, purse-shaped sponge, *Paragrantiawaguensis* is a cup or wide-mouthed tube.
- *Grantiacompressa* lacks a distinct fringe, *Paragrantiawaguensis* has a clear, differently colored, fringe containing special spicules with sizes not occurring in the rest of the body.
- Cortical skeleton of *Grantiacompressa* has club-shaped diactines in clusters, lacking in *Paragrantiawaguensis*
- Cortical skeleton of *Grantiacompressa* is thin and contains small triactines, *Paragrantiawaguensis* has a thick cortical skeleton including much larger triactines.
- Tubar triactines, subatrial triactines, atrial tri- and tetractines of *Grantiacompressa* are all smaller and thinner than those of *Paragrantiawaguensis*
- A special apopylar skeleton and spicules are lacking from *Grantiacompressa* and is the dominant feature in *Paragrantiawaguensis*

To date the World Porifera Database (Van Soest et al. 2015) lists 41 accepted species of the genus *Grantia* (including ‘Grantia’ waguensis). The accepted status of many of those species is uncertain, as there has been no recent revision of the genus and the names were taken more or less uncritically from Burton’s (1963) monograph. The most recent addition to the genus, *Grantia kempfi*, was made by Borojevic and Peixinho (1976). Among the species of *Grantia* s.l. there appears to be a wide variety of shapes and skeletal features, possibly divisible into distinct types, which may eventually lead to the distinction of subgenera or genera, leaving the genus name *Grantia* restricted to those species that share the properties of the above described *Grantia compressa*. Candidate species for such a restricted *Grantia* appear to be *Grantia cupula* (Haeckel, 1872), *Grantia extusarticulata* (Carter, 1886), *Grantia fistulata* Carter (1886), *Grantia foliacea* Breitfuss (1898), *Grantia stylata* Hôzawa (1929), *Grantia tenuis* Urban (1908), *Grantia aculeata* Urban (1908), *Grantia transgrediens* Brøndstedt (1931) and *Grantia uchidai* Hôzawa & Tanita (1941).

Other, not further specified groups of species may be distinguished e.g. on the possession of long protruding diactines (‘hairy’ *Grantia*’s), or those lacking tetractines, etc.

All these species do not have the apopylar specialization of *Paragrantia waguensis*. However, a few species assigned to the genus *Grantia* do appear to have at least a special category of atrial spicules, next to the usual atrial tri- and tetractines, viz. smaller tetractines in the apopylar region in *Grantia atlantica* Ridley (1881) as redescribed by Borojevic and Peixinho (1976), and in *Grantia nipponica* Hôzawa (1918). Furthermore, special apopylar spicules in the form of small diactines with serrated apices occur in *Grantia ramulosa* Dendy (1924). Possibly, these species could be united within *Paragrantia* by expanding its definition to include special apopylar spicules without specifying their shape. Such a decision is beyond the goals of the present study.

### Sequence data on *Paragrantia waguensis* and *Grantia compressa*

**Figure 8.**
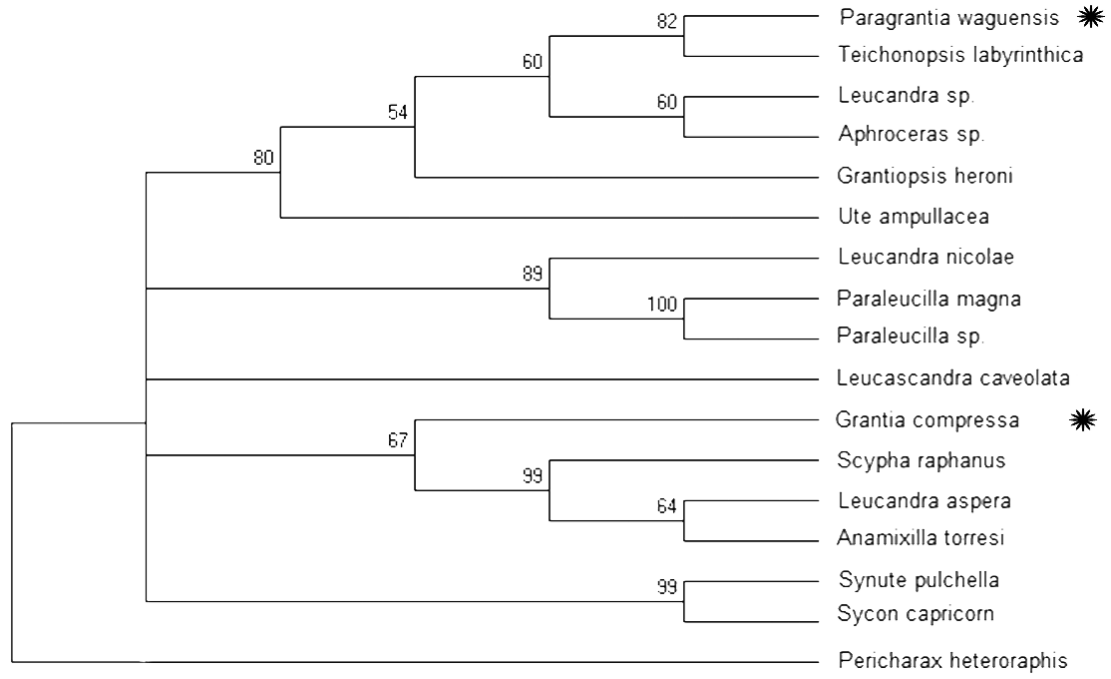
Phylogenetic tree (Maximum Likelihood, 50% majority consensus) of selected 28S partial sequences of calcareous sponge species belonging to the order Leucosolenida, including *Paragrantia waguensis* and *Grantia compressa* (asterisks). Sequences were downloaded from the NCBI website (http://www.ncbi.nlm.nih.gov/). The accession numbers and sources for the sequences are listed in Table 1. Bootstrap support values are based on 100 replicates. The species were selected on the basis of a BLAST search using a 430 bps 28S partial sequence of *Paragrantia waguensis* (specimen RMNH Por. 9317), provided by the Naturalis Biodiversity Center DNA laboratory. Sequences were aligned, trimmed and analyzed using MEGA 6.06 for Mac (http://www.megasoftware.net/megamac.php).

A further differentiation between the two type species of the genera *Paragrantia* and *Grantia* was obtained from sequences. The molecular classification of the Calcarea and its subclasses, orders and families is still in its early stages (see e.g. Voigt et al. 2012), so it is not straightforward to submit sequences and draw conclusions about likely affinity of various calcareous sponges. However, 28S sequences of *Grantia compressa* were submitted to Genbank by Manuel et al. (2004), so that gave us the opportunity to compare it with our new molecular data on *Paragrantia waguensis* (cf. above in the Methods section). We obtained from an analysis of 17 aligned Calcaronea sequences (see Table 1), using the program MEGA, a provisional phylogenetic tree (Maximum Likelihood, 50% majority consensus). The tree (cf. Fig. 8) shows moderately significantly that the two species compared here are only distantly related. *Paragrantia waguensis* was shown to have *Teichonopsis cylindrica* as its nearest relative, and *Grantia compressa* was retrieved in an isolated position near *Anamixilla*, *Scypha* (=*Sycon*) *raphanus* and *Leucandra aspera*.

## Conclusion

Until a revision of *Grantia* along the lines sketched above has been made – preferably guided by independent molecular markers of the studied taxa – we propose to maintain *Paragrantia* as a separate genus, so far monotypic with *Paragrantia waguensis* as the only species. Its status is comparable to other such genera (*Sycandra* and *Teichonopsis*), recognizable by unique features, as in this case the unique butterfly-shaped apopylar tetractines, for which we introduce the term ‘aliactines’ (from ala (L.) = wing).

We propose here the following definition (modified from Hôzawa 1940: 43):

**Genus Paragrantia Hôzawa, 1940**

**Type species.** *Paragrantia waguensis* Hôzawa, 1940 (by monotypy)

Syconoid Grantiidae with cortical skeleton of triactines, articulate tubar skeleton composed of aligned triactines, and an oscular fringe with giant diactines and sagittal tri-and tetractines. Atrial skeleton composed of subatrial triactines, and atrial triactines and/or tetractines. Choanocyte chambers connect with the atrial lumen through apopylar chambers lined with modified specialized tetractine spicules (aliactines).

## Supplementary Material

XML Treatment for Paragrantia waguensis
